# Effects of Key Environmental Factors on Growth of *Alternaria alternata* Isolated from Strawberry Jam and Its Production of Alternariol and Alternariol Monomethyl Ether

**DOI:** 10.3390/jof12050303

**Published:** 2026-04-22

**Authors:** Ju-Yeon Kim, Sung-Yong Hong, Ji-Su Kim, Ae-Son Om

**Affiliations:** Department of Food and Nutrition, College of Human Ecology, Hanyang University, Seoul 04763, Republic of Korea; au113rora@hanyang.ac.kr (J.-Y.K.); lunohong@hanyang.ac.kr (S.-Y.H.); ssen0825@hanyang.ac.kr (J.-S.K.)

**Keywords:** alternariol, alternariol monomethyl ether, *Alternaria alternata* OM1, pH, relative humidity, temperature

## Abstract

Alternariol (AOH) and alternariol monomethyl ether (AME) are major mycotoxins produced primarily by *Alternaria alternata* on cereal grains and fruits. *A. alternata* is a causative pathogen of strawberry black spot disease. However, little is known about the characteristics of *A. alternata*, which was isolated from strawberry products. In the present study, we evaluated the influence of temperature, pH, and relative humidity (RH) on the growth of *A. alternata* OM1 and its production of AOH and AME on different media including strawberry puree agar medium (SPAM) after its isolation from strawberry jam. The fungal strain showed the highest growth rate at 25 °C under pH 6.5 and RH 97%, while the highest amounts of AOH and AME were produced by the strain at 25 °C under pH 4.5 and RH 97%. Additionally, the strain did not produce AOH and AME on SPAM at 25 °C under RH 92% until 7 days. Moreover, RT-qPCR analysis exhibited that relative expression levels of 2 AOH or AME biosynthetic genes (*pksI* and *omtI*) in *A. alternata* OM1 were up-regulated in YES medium, while they were not in MEB medium. Our results demonstrated that the three key environmental parameters had a significant influence on the growth of *A. alternata* OM1 and its production of AOH and AME. These findings suggest that storage of strawberries below 25 °C under RH 92% could prevent the production of AOH and AME by *A. alternata* OM1 on them.

## 1. Introduction

*Alternaria* species are ubiquitous in soil, and most of them are both saprophytic and pathogenic on various plants such as cereal grains, fruits, and vegetables [1,2,3]. Some species of *Alternaria*, including *Alternaria alternata*, are well-known for the production of toxic secondary metabolites on wheat grains, apples, and tomatoes during postharvest and storage [4,5,6]. In particular, *A. alternata* is the most common species found in fruits and vegetables, and is one of the main pathogens that cause strawberry black spot disease [7,8,9]. This fungus is able to produce two dibenzopyrone derivative toxins, alternariol (AOH) and alternariol monomethyl ether (AME), and one tetramic acid derivative toxin, tenuazonic acid (TeA), as the main *Alternaria* toxins on cereal grains and fruits including strawberries [4,5,6,10,11]. Although the three major mycotoxins have relatively low acute toxicities, it has been documented that they produce cytotoxic, genotoxic, mutagenic, and carcinogenic effects on mammalian cells [4,5,6,12,13]. In particular, of the three major toxins, AOH exhibited more genotoxic properties than AME in human colon carcinoma cells and was able to induce squamous cell carcinoma in human embryo esophageal tissues [4]. Also, it is known that AOH and AME have endocrine-disrupting properties because of their structural similarity to genistein, which is an estrogen-like compound in soybeans [5,14], and that they exhibit synergistic effects [15]. In addition, these mycotoxins are relatively thermostable during food processing [5,16]. Nevertheless, since there are insufficient data on their toxicities and human exposure, the regulatory limits on food and food-derived products were not established [17]. Thereafter, a toxicological review by the European Food Safety Authority (EFSA) suggested that the main *Alternaria* toxins are of great concern in public health [18]. Recently, the European Commission (EC) has set the recommended levels of the three major *Alternaria* toxins on certain food: 10 μg/kg of AOH, 5 μg/kg of AME, and 500 μg/kg of TeA for processed tomato products [19].

Previously, it has been reported that several environmental parameters, such as temperature, pH, and relative humidity (RH), can affect both fungal growth and mycotoxin production on food and food-derived products [20,21]. To date, most of the previous studies, which were related to environmental conditions for the main *Alternaria* toxin production, have used synthetic or semi-synthetic media or natural food products for cultures of *A. alternata* or *Alternaria arborescens* strains, which were isolated from soybeans, chickpeas, wheat grains, tomatoes, grapes, and cherries [2,3,11,22,23,24,25,26,27,28]. However, there are no studies on the production of the main mycotoxins on strawberry-based medium by *Alternaria* sp. isolated from strawberries or strawberry products. Thus, in the present study, we isolated *A. alternata* OM1 from strawberry jam, verified its AOH and AME production by LC/MS/MS, and cultured the fungal strain under different temperature, pH, and RH conditions to find the optimum conditions for its growth and production of AOH and AME on natural product media containing strawberry puree, which is a simulated natural environment using strawberries. Additionally, we evaluated the relative expression levels of 3 AOH or AME biosynthetic genes (*pksI*, *omtI*, and *aohR*) in *A. alternata* OM1 under AOH-conducive and non-conducive synthetic media. To the best of our knowledge, this is the first report on the effects of the three key environmental parameters on the production of AOH and AME by *A. alternata* on natural product media containing strawberry puree. Our findings in the current study could be helpful for the development of potential methods to efficiently control the growth of *A. alternata* and its production of AOH and AME on fresh fruits such as strawberries.

## 2. Materials and Methods

### 2.1. Isolation of Fungi from Strawberry Jam

Three jars of homemade strawberry jam, which were made of one strawberry cultivar (Sulhyang), were collected at the Yangpyeong Agricultural Technology Service Center (ATST; Yangpyeong, Gyeonggi, Republic of Korea) in the spring of 2023, and 3 g of jam from each jar was used to search for AOH-producing fungi. After the collected strawberry jam samples were diluted with 0.85% sterile saline solution, the suspension was inoculated onto potato dextrose agar (PDA; MB Cell, Seoul, Republic of Korea) plates containing 2 different types of antibiotic solutions (both 1 mg of chloramphenicol and 1 mg of tetracycline in 200 mL of PDA). They were then incubated at 30 °C for 4 days. For isolation of pure fungal strains, each fungal isolate was transferred onto a new PDA plate and incubated at 30 °C for 4 days. Next, for fungal spore preparation, after 0.01% Tween 80 solution was added onto each agar plate, the fungal spores were scraped off using a sterile microspatula (SUS304, NAVIMRO, Seoul, Republic of Korea), filtered through 3 layers of sterilized cheesecloth, and collected by centrifugation at 8000 rpm for 15 min. After the spore preparation by resuspending the spores in 20% sterilized glycerol, pure fungal strains were isolated by inoculation of diluted spore suspension onto PDA plates, which was repeated by three rounds of isolation.

### 2.2. Identification of Fungal Isolates and Phylogenetic Analysis

For microscopic observation of morphological structures, after spore preparation with 0.01% Tween 80 solution from each fungal isolate grown on PDA agar plates, fungal spores (10 μL of 2 × 10^7^ spores/mL) were inoculated onto the center of each PDA or malt extract agar (MEA; MB Cell, Seoul, Republic of Korea) plate and incubated at 30 °C for 5 days. The morphological structure of each fungal isolate was then observed using a lactic acid slide mount under a microscope (Olympus CHK2-F-GS, Olympus Co. Ltd., Tokyo, Japan).

For molecular identification, fungal isolates were analyzed by DNA sequencing of 3 regions (internal transcribed spacer [ITS], calmodulin [CaM], β-tubulin [BenA]) on fungal DNA [29,30,31]. For genomic DNA isolation, after spore preparation with 0.01% Tween 80 solution from fungal isolates grown on PDA plates, fungal spores (0.5 mL of 2 × 10^7^ spores/mL) were inoculated into 100 mL of potato dextrose broth (PDB; MB Cell, Seoul, Republic of Korea) in a 250 mL flask and incubated at 30 °C for 5 days with shaking at 150 rpm. Genomic DNA was isolated from fungal mycelia by a protocol of Steven B Lee and John W. Taylor using phenol/chloroform/isoamyl alcohol (25:24:1, PCI; Biochemicals Inc.; Gyeonggi, Republic of Korea) with some modifications (use of 3 M sodium acetate [pH 5.2] and 50 mM Tris-HCl [pH 8.0]) [32] as described previously [29]. Next, in order to identify fungal species, 3 regions (ITS, CaM, and BenA) of the isolated genomic DNA were amplified by polymerase chain reaction (PCR) along with a set of universal primers for each region as described previously [29,30,31,33]. The primer sequences are as follows: ITS1 (5′-TCCGTAGGTGAACCTGCGG-3′, forward) and ITS4 (5′-TCCTCCGCTTATTGATATGC-3′, reverse) for ITS region, Cmd5 (5′-CCGAGTACAAGGAGGCCTTC-3′, forward) and Cmd6 (5′-CCGATAGAGGTCATAACGTGG-3′, reverse) for CaM region, and Bt2a (5′-GGTAACCAAATCGGTGCTGCTTTC-3′, forward) and Bt2b (5′-ACCCTCAGTGTAGTGACCCTTGGC-3′, reverse) for BenA region. PCR was performed at 95 °C for 5 min, followed by 35 cycles of denaturation at 95 °C for 1 min, annealing at 55 °C (for ITS), 54 °C (for CaM), or 57 °C (for BenA) for 1 min, and an extension at 72 °C for 2 min, and a final extension at 72 °C for 10 min. After PCR products were separated on 1.2% (*w*/*v*) agarose gels by electrophoresis, approximately 500–800 bp of PCR products were purified using AccuPep PCR/Gel Purification Kit (Bioneer, Daejeon, Republic of Korea) and sequenced at Biofact Co. (Daejeon, Republic of Korea). Then, the fungal isolates were identified by the local similarity between nucleotide sequences of the PCR products and nucleotide sequences of the same region in fungal strains retrieved from GenBank in the National Center for Biotechnology Information (NCBI) using nucleotide Basic Local Alignment Search Tool (BLASTn; https://blast.ncbi.nlm.nih.gov/Blast.cgi; 15 June 2023).

The phylogenetic tree was constructed using the Molecular Evolutionary Genetics analysis (MEGA) program (v. 11), which was based on the neighbor-joining (NJ) method, and nucleotide sequences of identified fungal species [34].

### 2.3. Fungal Culture Conditions

After spore preparation with 0.01% Tween 80 solution as described above, fungal spores (100 μL of 10^7^ spores/mL) were inoculated into 5 mL of PDB and incubated at 30 °C for 5 days with shaking at 120 rpm to screen for AOH-producing fungal strains by high-performance liquid chromatography (HPLC; see below).

The following experiments were performed with one fungal strain isolated as an AOH producer from strawberry jam.

For fungal culture on strawberry puree agar medium (SPAM; strawberry without stems:distilled water [DW] = 80:20, *w*/*w*) containing homogenized strawberry (cultivar Sulhyang) after homogenization of strawberry without stems at 5000 rpm for 10 min using a Daihan Scientific homogenizer (HG-15D; Seoul, Republic of Korea), yeast extract sucrose (YES; 2% yeast extract, 15% sucrose, 0.1% MgSO_4_·H_2_O), and MEA agar plates, 10 μL of 10^7^ spores/mL were center-inoculated onto each plate, which was overlaid with a sterile cellophane membrane (Sigma-Aldrich Co.; St. Louis, MO, USA). The agar plates were then incubated for 21 days at 4 different temperatures (5, 20, 25, and 30 °C).

For fungal culture under 4 different pH conditions (4.5, 5.5, 6.5, and 7.5) to analyze its growth, SPAM (pH 3.8, unadjusted pH), YES, or MEA plates were prepared after the pH of the media was adjusted by adding HCl or NaOH. The agar plates were then incubated at 25 °C for 21 days after inoculation as described above. Also, to analyze the levels of AOH and AME produced by the fungal strain, SPAM, YES, or MEA plates (pH 4.5, 5.5, and 6.5) were incubated at 20, 25, and 30 °C for 21 days after inoculation of spores.

For fungal culture under different RH conditions, SPAM (pH 4.5) plates were placed in sealed plastic containers (200 mm × 150 mm × 80 mm; Daiso, Seoul, Republic of Korea; 6 SPAM plates in one container), which were adjusted to approximately 92 or 97% RH using saturated salt solutions (KNO_3_ for 92% RH and K_2_SO_4_ for 97% RH at 25 °C) [35]. The containers were then incubated at 25 °C for 14 days.

For microscopic observation of morphological structures of the fungal strain grown on 4 different agar plates, fungal spores (10 μL of 2 × 10^7^ spores/mL) were center-inoculated onto each SPAM, PDA, YES, and MEA plate and incubated at 25 °C for 5 days. Then, the morphological structures were observed using lactic acid slide mounts under a microscope (Olympus IX 71, Olympus Co. Ltd., Tokyo, Japan).

For liquid culture, fungal spores (1 mL of 10^7^ spores/mL) were inoculated into 100 mL of YES (pH 4.5) or MEB (pH 4.5) in a 250 mL flask, and incubated at 25 °C for 10 days under shaking and no-shaking conditions at 150 rpm.

For evaluation of TeA production by the fungal strain under culture media containing dimethyl sulfoxide (DMSO; Sigma-Aldrich Co.; St. Louis, MO, USA), fungal spores (100 μL of 10^7^ spores/mL) were inoculated into 5 mL of PDB or PDB supplemented with DMSO at a 1 or 5% (*v*/*v*) concentration after filtration of DMSO with a 0.2 μm sterile syringe filter and incubated at 25 °C for 5 days under static conditions.

For relative expression analysis of AOH or AME biosynthetic genes in the cluster by RT-qPCR, fungal spores (1 mL of 10^6^ spores/mL) were inoculated into 100 mL of YES or MEB in a 250 mL flask, and incubated at 25 °C for 10 days under no shaking (for YES) or shaking conditions (for MEB) at 150 rpm.

### 2.4. Measurement of Fungal Growth

Fungal growth was analyzed by measuring the dry weight of mycelia and the diameter of colonies grown on SPAM, YES, and MEA plates. Mycelial dry weight was assessed by complete drying of the entire mycelia on cellophane membranes, which had covered SPAM, YES, and MEA plates, at 80 °C. Radial mycelial growth was determined by measuring the two diameters of the colony at 90° angles to each other [21]. The mycelial dry weight and colony diameter were measured in triplicate.

### 2.5. AOH, AME, and TeA Extraction

AOH, AME, and TeA extraction were performed by the method described by Patriaca et al. with slight modifications [15]. Briefly, each culture was extracted with 30 mL methanol (MeOH; ≥98.0% purity; Avantor Performance Materials Inc., Radnor, PA, USA) by shaking for 1 h using a Wrist Action Shaker (Burrell Scientific, Pittsburgh, PA, USA). After the extract was filtered through glass microfiber (GF/A) filter paper (Whatman, Maidstone, UK) and clarified with 20% ammonium sulfate (Junsei Chemical Co., Tokyo, Japan), it was divided into two parts for two separate extractions (AOH plus AME, TeA). One part was extracted twice with 10 mL of chloroform (98.0% purity; Samchun Chemical Co., Seoul, Republic of Korea). The organic phases were collected, combined, and evaporated to dryness under a gentle stream of nitrogen at 40 °C. The residue was dissolved in 4 mL MeOH and filtered through a 0.2 μm polyvinylidene fluoride (PVDF) syringe filter (Hyundai Micro Co., Seoul, Republic of Korea) for AOH and AME analysis by HPLC. The other part was adjusted to pH 2 with 6 N HCl. It was extracted twice with 15 mL of chloroform. After the addition of 10 mL of 5% Sodium bicarbonate (NaHCO_3_; Samchun Chemical Co., Seoul, Republic of Korea), the aqueous phase was acidified to pH 2 again and extracted twice with 10 mL of chloroform. The extract was washed with 7.5 mL of DW and dried under nitrogen at 40 °C. The residue was dissolved in 4 mL MeOH and filtered through a 0.2 μm PVDF syringe filter for TeA analysis by HPLC.

### 2.6. Preparation of AOH, AME, and TeA Standard Solutions and HPLC Analysis

An AOH or AME stock solution (200 μg/mL) was prepared by dissolving 5 mg of AOH or AME powder (≥98.0% purity; Sigma-Aldrich Co.; St. Louis, MO, USA) in 25 mL of MeOH and stored at −20 °C until use. Five levels of AOH or AME standard solutions (0.1, 0.2, 0.5, 1.0, and 2.0 μg/mL) were freshly made by diluting each stock solution with MeOH. In order to prepare a TeA stock solution (1000 μg/mL), 10 mg of TeA powder (≥98.0% purity; Sigma-Aldrich Co.; St. Louis, MO, USA) was dissolved in 10 mL of MeOH and stored at −20 °C until use. A series of TeA standard solutions (0.5, 1.0, 2.0, 5.0, and 10.0 μg/mL) were freshly prepared by dilutions of the stock solution with MeOH.

The three types of *Alternaria* toxins (AOH, AME, and TeA) were analyzed by an HPLC system (LC-20AT, Shimadzu; Tokyo, Japan) equipped with UVD (SPD-10A, Shimadzu; Tokyo, Japan). The determination of AOH and AME was carried out at 258 nm, whereas that of TeA was carried out at 280 nm. Separation of analytes was performed on a ZORBAX Eclips plus C18 column (5 μm particle size, 4.6 mm × 250 mm, Agilent; Santa Clara, CA, USA). For AOH and AME quantification, the mobile phase consisted of 80% MeOH (MeOH:DW = 80:20, *v*/*v*) containing 300 mg of zinc sulfate (ZnSO_4_·H_2_O; Sigma-Aldrich Co.; St. Louis, MO, USA)/L of solvent, while for TeA quantification, the mobile phase was composed of 85% MeOH (MeOH:DW = 85:15, *v*/*v*) containing 300 mg of zinc sulfate (ZnSO_4_·H_2_O)/L of solvent. The mobile phase was pumped into the HPLC system at a flow rate of 0.4 mL/min, giving a total run time of 25 min for AOH and AME detection, and 20 min for TeA detection. The injection volume of samples was 10 μL and the column oven temperature was set at 30 °C.

The linearity of a series of AOH, AME, or TeA concentrations in the HPLC analytical method was assessed by a calibration curve using AOH, AME, or TeA standard solutions (0.1, 0.2, 0.5, 1.0, and 2.0 μg/mL for AOH and AME, 0.5, 1.0, 2.0, 5.0, and 10.0 μg/mL for TeA). The calibration curve of AOH, AME, or TeA was created by plotting the peak areas (y axis) versus AOH, AME, or TeA concentrations (x axis) in HPLC-UVD analyses. The linearity was evaluated by linear regression analysis and determined by a coefficient of determination (r^2^). The r^2^ value of the AOH, AME, or TeA standard curves was 0.999 (Figure S1).

A limit of detection (LOD) and limit of quantification (LOQ) were used to determine the sensitivity of the HPLC-UVD analytical method. They were calculated using the slope (S) of the standard curve and the standard deviation (SD) of the response, which were obtained from linearity assessment, as follows:

(1) $$LOD=\frac{3.3\times SD}{\begin{array}{c} S \end{array}}$$

(2) $$LOQ=\frac{10\times SD}{S}$$

The LOD and LOQ for AOH were 0.032 and 0.097 μg/mL, while those for AME were 0.015 and 0.046 μg/mL, respectively. Also, the LOD and LOQ for TeA were 0.024 and 0.073 μg/mL, respectively.

### 2.7. LC-MS/MS Analysis

Liquid chromatography-tandem mass spectrometry (LC-MS/MS) was used to confirm the identity of AOH and AME extracted from liquid culture. The LC-MS/MS analysis was carried out using an Agilent 1290 Infinity UHPLC system (Santa Clara, CA, USA), which was connected to an Agilent 6545XT LC/Quadrupole Time-of-Flight (Q-TOF) (Santa Clara, CA, USA) LC/MS equipped with a dual-spray Agilent Jet Stream (AJS) electrospray ionization (ESI) source. Separation of analytes was performed on an Agilent ZORBAX Eclipse Plus C18 column (1.8 μm particle size, 100 mm × 2.1 mm; Santa Clara, CA, USA) through an online mixture of solvent A (100% pure water) and solvent B (100% MeOH) as a mobile phase with a flow rate of 0.25 mL/min. The injection volume of samples was 20 μL and the column oven temperature was maintained at 30 °C. An applied gradient elution program is as follows: after the B solution was constantly increased to 5% from 0 min to 1 min, it continuously increased to 60% from 1 min to 3 min. Then, it linearly increased to 95% from 3 min to 10 min and was held on 95% until 17 min. Subsequently, the 95% B solution rapidly decreased from 95% at 17 min to 5% at 18 min, and the 5% B solution was maintained for 1 min for re-equilibration of the column before injection of the next sample.

The general MS parameters in the negative mode were set as follows: capillary voltage, 4000 V; nozzle voltage, 500 V; drying gas temperature, 130 °C; drying gas flow rate, 6 L/min; nebulizer gas pressure, 30 psi; sheath gas temperature, 300 °C; sheath gas flow rate, 11 L/min. The peak spectrum was acquired using the Find by Formula data-mining algorithm. Data were processed using Agilent MassHunter Qualitative Analysis Software (rev. 10.0; Santa Clara, CA, USA).

### 2.8. Relative Gene Expression Analysis by RT-qPCR

After the fungal strain was cultured in YES under static conditions or MEB under agitation conditions, the harvested mycelia were immediately frozen in liquid nitrogen for total RNA isolation. Additionally, after AOH or AME was extracted from culture filtrates, the amounts of the toxins in the extract were quantified by HPLC analysis as described above.

Total RNA isolation was carried out with mycelia using the RNeasy Mini Kit (QIAGEN, Hilden, Germany) following a procedure provided by the manufacturer. The RNA quality was evaluated by Agilent 2100 Bioanalyzer (Agilent Technologies, Santa Clara, CA, USA).

Specific primer pairs of AOH or AME biosynthetic genes were designed, based on the genome sequence of *A. alternata* OM1, using Primer3Plus (https://www.primer3plus.com/index.html; 20 January 2025) (Table 1) [36]. The specificity of the primer sequences was assessed using the Primer-BLAST online tool (https://www.ncbi.nlm.nih.gov/tools/primer-blast/; 20 January 2025) at NCBI [37,38]. The β-tubulin gene of *A. alternata* NSH-1 (GenBank accession number MN175551) was used to design a pair of specific primers for an internal control [39].

The cDNA synthesis was conducted using PrimerScript RT Reagent Kit with gDNA Eraser (Perfect Real Time) from TaKaRa (Takara Bio Inc., Shiga, Japan) following the instructions provided by the manufacturer as described previously [29].

For RT-qPCR amplification, TB Green Premix Ex Taq II (Tli RNaseH Plus) ROX plus Kit (Takara Bio Inc., Shiga, Japan) was used, and the reactions were carried out according to the manufacturer’s instructions as described previously [29]. RT-qPCR reactions were performed at 95 °C for 2 min, followed by 40 cycles of denaturation at 95 °C for 5 s, annealing at 58 °C for 30 s, and extension at 72 °C for 5 s. After the final amplification cycle, a melting curve analysis of the PCR products was conducted by a 0.5 °C increase per sec from 60 to 95 °C to verify the formation of a single PCR product. The data were analyzed by the relative quantification method using CFX Maestro software (v. 4.1; Bio-Rad, Hercules, CA, USA). The relative gene expression level was calculated by the 2^−ΔΔCT^ method (comparative CT method) [40]. The β-tubulin gene was used as an internal control for normalization. The RT-qPCR analysis was carried out with three independent biological replicates, and two duplicates were analyzed for each biological sample.

### 2.9. Statistical Analyses

Data were statistically analyzed by a one-way or two-way analysis of variance (ANOVA), which was followed by Duncan’s test as a post hoc analysis. The data were represented as the mean ± SD using SPSS 26.0 (SPSS Inc., Chicago, IL, USA). A *p* value < 0.05 was considered statistically significantly different. All analyses from fungal cultures were carried out in triplicate.

## 3. Results

### 3.1. Isolation of Fungi from Strawberry Jam, Identification of the Isolated Fungi, and Analyses of AOH, AME, or TeA Production by the Isolated Fungi

**Figure 1:**
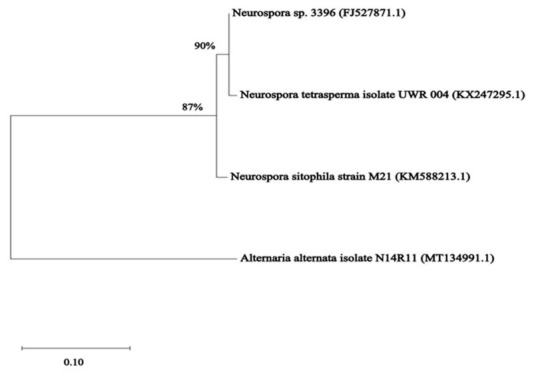
Phylogenetic relationship based on sequences of ITS rDNA region from fungi isolated from strawberry jam. The trees were constructed using the neighbor-joining (NJ) method.

**Figure 2:**
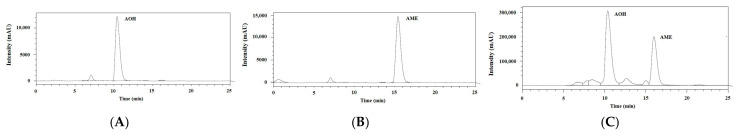
HPLC chromatograms of AOH and AME. Chromatograms of (**A**) AOH standard solution (2 μg/mL), (**B**) AME standard solution (2 μg/mL), and (**C**) extracts from culture of fungal isolate A2 (*A. alternata*) for AOH and AME analyses. AOH and AME were eluted at 10.4 and 15.4 min, respectively.

A total of five fungi were isolated from strawberry jam. Then, the morphological characteristics of the five fungal isolates, such as their conidiospores and conidiophores, were observed under the microscope after culture on PDA and MEA agar plates. Next, the five fungal isolates were identified genetically based on sequences of the ITS1-5.8S rDNA-ITS2 region on fungal rDNA. When the sequences of the ITS region in PCR products were compared with those of the same region from reference strains in the GenBank database at the NCBI website using BLASTn, the analysis exhibited high degrees of sequence similarity (98–100%) between the nucleotide sequences of the fungal isolates and the reference strains. In addition, for more accurate fungal identification, we performed another PCR using two pairs of specific primers for amplification of two other DNA regions (CaM and BenA). The identification results were identical to the ITS region-based ones. One out of five isolates was identified as *A. alternata*, while four isolates belonged to *Neurospora* spp. Of four isolates, two were identified as the same strain (*Neurospora sitophila*), whereas one isolate was identified as *Neurospora tetrasperma* (Table S1). The phylogenetic tree based on ITS sequences from the five fungal isolates is shown in Figure 1. This result indicates that strawberry jam was contaminated with fungi that belong to two genera: *Neurospora* spp. (three species, except one unknown species) and *Alternaria* spp. (one species). The four fungal species were further categorized into two orders (*Neurospora* spp. in Sordariales, *Alternaria* spp. in Pleosporales). Next, the five fungal isolates were screened for AOH production by HPLC analysis. Of the five fungal isolates, one isolate (A2, *A. alternata*) was able to produce moderate levels of AOH (4.67 ± 0.44 μg/mL) and AME (1.33 ± 0.14 μg/mL) (Table S1 and Figure 2). We then named the AOH-producing *A. alternata* isolate (A2) *A. alternata* OM1. The morphology of *A. alternata* OM1 on 4 types of agar media (PDA, SPAM, YES, and MEA) is shown in Figure S2. Conidiospores of the strain showed typical light brown obclavate (inverse club-shaped) characteristics of *Alternaria* spp. (Figure S2E,F).

The identity of AOH and AME produced by *A. alternata* OM1 was confirmed by LC-MS/MS analysis. The AOH standard and that in the fungal culture extracts were eluted at 6.596 min and 6.618 min, respectively (Figure S3A,C). The mass-to-charge (*m*/*z*) ratios of the most abundant product ion [M-H]^−^ associated with the peaks in the AOH standard and the fungal culture extracts were almost identical (257.0462 and 257.0455, respectively), and the MS profiles were a close match (Figure S3B,D). In addition, we found another substance in the fungal culture extracts, which was eluted at 8.369 min (Figure S3G). The *m*/*z* ratio of the most abundant product ion [M-H]^−^ associated with the substance peak was 271.0616, and its molecular formula was C_15_H_12_O_5_ (Figure S3H). The AME standard was also eluted at 8.336 min and the *m*/*z* ratio of the most abundant product ion [M-H]^−^ associated with the peak in the AME standard was 271.0618 (Figure S3E,F), which was almost identical with that of the substance detected in the fungal extracts (*m*/*z* = 271.0616). These results confirmed that *A. alternata* OM1 produced AOH and AME.

Since *A. alternata* can usually produce TeA as well as AOH and AME, we attempted to analyze the culture extracts of *A. alternata* OM1 to see whether the fungal strain is able to produce TeA. However, the strain was not able to produce TeA in PDB or PDB supplemented with DMSO.

### 3.2. Influence of Temperature and Culture Media on the Growth of A. alternata OM1

**Figure 3:**
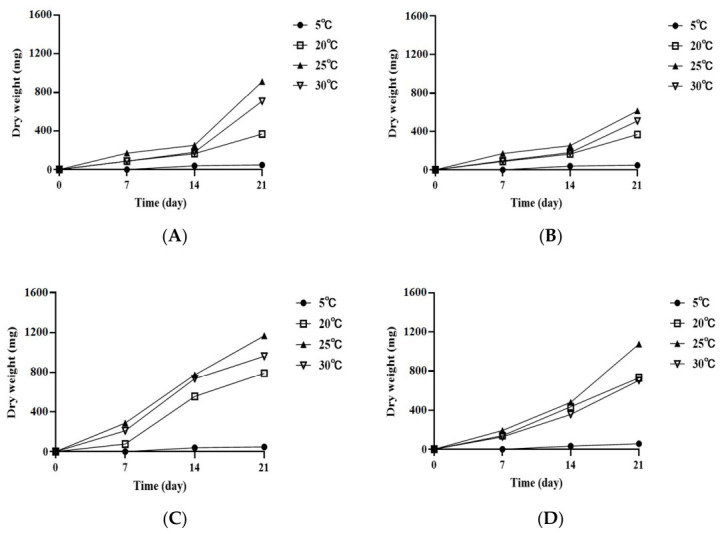
Growth rates of *A. alternata* OM1 on four types of solid media (SPAM, PDA, YES, and MEA) under four different temperature conditions (5, 20, 25, and 30 °C). (**A**) Dry weight of mycelia on SPAM, (**B**) dry weight of mycelia on PDA, (**C**) dry weight of mycelia on YES, and (**D**) dry weight of mycelia on MEA. The mycelial dry weight was measured in triplicate. Data are represented as the mean ± standard deviation.

To evaluate the influence of temperature and culture media on the growth of *A. alternata* OM1, the fungal strain was cultured on 4 different types of solid media (SPAM, PDA, YES, and MEA) under 4 different temperature conditions (5, 20, 25, and 30 °C). The fungal growth showed similar trends between SPAM and PDA or between YES and MEA (Figure 3). The growth rates on SPAM and PDA increased slowly until 14 days of incubation, and thereafter they slightly rapidly increased at 20, 25, and 30 °C until 21 days (Figure 3A,B). In contrast, the growth rates on YES and MEA increased relatively constantly under the three temperature conditions over 21 days (Figure 3C,D). The fungal growth was highest on YES (dry weight, 1169.10 ± 79 mg) at 25 °C after 21 days of incubation, which was followed by MEA (dry weight, 1075.73 ± 511 mg), SPAM (dry weight, 912.40 ± 48 mg), and PDA (dry weight, 612.43 ± 48 mg). In addition, the fungal strain exhibited the highest growth at 25 °C on all 4 different types of media after 21 days of incubation, followed by 30 and 20 °C, although the growth rates on MEA at the latter two temperatures did not show a statistically significant difference from each other. However, almost no growth was observed on all 4 types of media at 5 °C over 21 days of incubation. The fungal growth based on the colony diameter showed similar patterns to that based on the dry weight with slight differences (Figure S4). The fungal strain produced similar radial growth rates on YES and MEA (Figure S4C,D). The growth on the two media approached a plateau at 20 and 25 °C after 14 days of incubation, although the rate at 30 °C increased until the 21st day. In contrast, the fungal growth on SPAM increased relatively constantly under the three temperature conditions over 21 days (Figure S4A). The rates on YES were slightly better than those on SPAM, and the growth on both media was higher than that on MEA at 25 °C over 21 days (9.00 ± 0.00 cm on YES, 9.00 ± 0.00 cm on SPAM, 7.74 ± 0.02 cm on MEA after 21 days). However, the growth rates on PDA were significantly retarded under the three temperature conditions compared to those on the three media (Figure S4B). Also, although the radial growth rates on all four different types of media were highest at 25 °C, there was no radial growth on all types of media at 5 °C over 21 days. It is different from the growth based on the mycelial dry weight, which increased slightly on the same types of media at 5 °C after 7 days (Figure 3 and Figure S4).

Overall, these results indicate that *A. alternata* OM1 on four different types of media had an optimum growth temperature at 25 °C. The fungal strain also showed the highest growth rate on YES at 25 °C after 21 days of incubation.

### 3.3. Influence of pH and Culture Media on the Growth of A. alternata OM1

**Figure 4:**
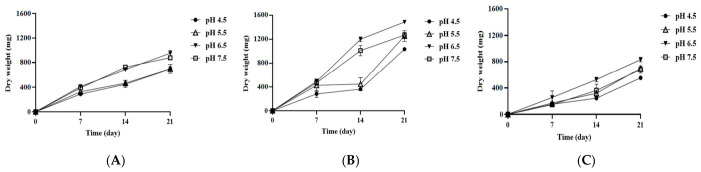
Growth rates of *A. alternata* OM1 on three types of solid media (SPAM, YES, and MEA) under four different pH conditions (pH 4.5, 5.5, 6.5, and 7.5) at 25 °C. (**A**) Dry weight of mycelia on SPAM at 25 °C, (**B**) dry weight of mycelia on YES at 25 °C, and (**C**) dry weight of mycelia on MEA at 25 °C. The mycelial dry weight was measured in triplicate. Data are represented as the mean ± standard deviation.

It has been documented that environmental pH influences fungal growth by disrupting intracellular enzyme activity due to pH changes inside cells [41]. Thus, in order to investigate the optimal pH for the growth of *A. alternata* OM1, the fungal strain was cultured on three types of solid media (SPAM, YES, and MEA) under four different pH conditions (pH 4.5, 5.5, 6.5, and 7.5) at 25 °C. The fungal growth increased steadily on all three types of media under all four pH conditions until 21 days of incubation (Figure 4). The growth on all three types of media was highest at pH 6.5 among all four pH conditions, which was followed by pH 7.5, 5.5, and 4.5 after 21 days of incubation. The fungal strain also exhibited the highest growth on YES (pH 6.5) among the three different media after 21 days (dry weight, 1486.40 ± 8 mg). In addition, the fungal growth based on the colony diameter showed similar trends to that based on the dry weight (Figure S5). The radial growth rates increased gradually on all three types of media under all four pH conditions over 21 days although they approached a plateau at pH 6.5 and 7.5 from the 14th day to the 21st day (Figure S5). The fungal strain already reached the highest growth on YES at pH 6.5 and 7.5 after the 14th day (colony diameter, 9.00 ± 0.00 mg), which is similar to the results based on the above dry weight. These results indicate that *A. alternaria* OM1 had the optimum pH of pH 6.5 for its growth at 25 °C. When taken together with the above results, the fungal strain showed the highest growth on YES under pH 6.5 and 25 °C conditions.

### 3.4. Influence of the Combination of pH and Temperature on Production of AOH and AME by A. alternata OM1 on Three Types of Culture Media

**Figure 5:**
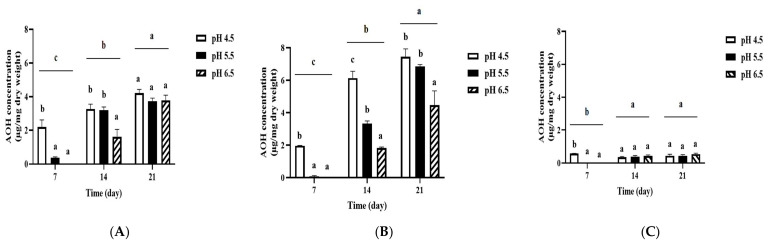

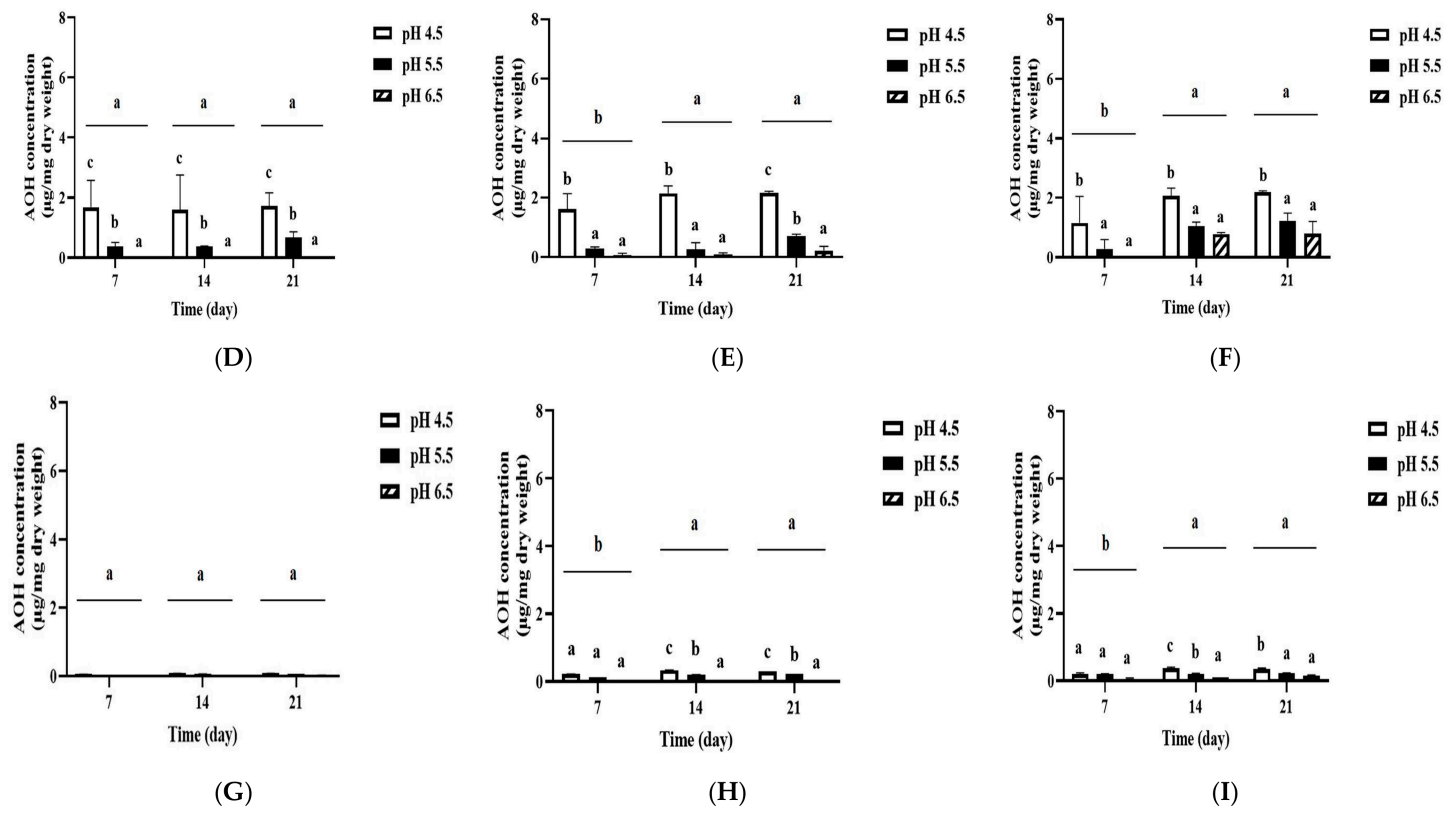
AOH production by *A. alternata* OM1 on three types of solid media (SPAM, YES, and MEA) under the combination of three different temperatures (20, 25, and 30 °C) and three different pH conditions (pH 4.5, 5.5, and 6.5). (**A**) Levels of AOH on SPAM at 20 °C, (**B**) levels of AOH on SPAM at 25 °C, (**C**) levels of AOH on SPAM at 30 °C, (**D**) levels of AOH on YES at 20 °C, (**E**) levels of AOH on YES at 25 °C, (**F**) levels of AOH on YES at 30 °C, (**G**) levels of AOH on MEA at 20 °C, (**H**) levels of AOH on MEA at 25 °C, and (**I**) levels of AOH on MEA at 30 °C. The levels of AOH were measured in triplicate. Data are represented as the mean ± standard deviation. Different letters indicate statistically significant differences (*p* < 0.05).

It has been reported that environmental pH also influences mycotoxin production by changes in the expression levels of mycotoxin biosynthetic genes [42,43]. Thus, we analyzed the amounts of AOH and AME produced by *A. alternata* OM1 on three types of solid media (SPAM, YES, and MEA) under the combination of three different temperatures (20, 25, and 30 °C) and three different pH conditions (pH 4.5, 5.5, and 6.5). Unlike the above results of the fungal growth, the strain produced the highest levels of AOH on all three types of media at pH 4.5 after 21 days of incubation, which was followed by pH 5.5 and 6.5 (Figure 5). In addition, the level of AOH production on all three types of media was highest at 25 °C after 21 days, which is similar to the fungal growth pattern as described above, although the levels showed a plateau after 14 days on all types of media except on SPAM at 20 and 25 °C. Also, the strain produced approximately 3.5-fold higher levels of AOH on SPAM (7.46 ± 0.47 μg/mg dry weight) than on YES (2.17 ± 0.03 μg/mg dry weight) under pH 4.5 and 25 °C conditions after 21 days of incubation, whereas it produced much lower levels of AOH on MEA (0.29 ± 0.01 μg/mg dry weight) than on SPAM or YES under the same conditions. When the incubation temperature increased to 30 °C from 25 °C, AOH production on SPAM was significantly reduced (Figure 5B,C). These results imply that the fungal strain produced the highest amount of AOH on SPAM under pH 4.5 and 25 °C conditions.

**Figure 6:**
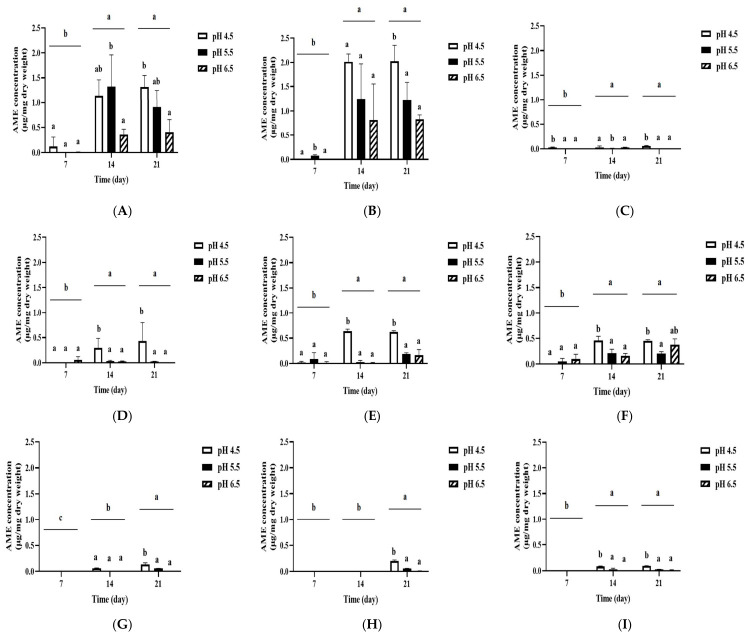
AME production by *A. alternata* OM1 on three types of solid media (SPAM, YES, and MEA) under the combination of three different temperature (20, 25, and 30 °C) and three different pH conditions (pH 4.5, 5.5, and 6.5). (**A**) Levels of AME on SPAM at 20 °C, (**B**) levels of AME on SPAM at 25 °C, (**C**) levels of AME on SPAM at 30 °C, (**D**) levels of AME on YES at 20 °C, (**E**) levels of AME on YES at 25 °C, (**F**) levels of AME on YES at 30 °C, (**G**) levels of AME on MEA at 20 °C, (**H**) levels of AME on MEA at 25 °C, and (**I**) levels of AME on MEA at 30 °C. The levels of AME were measured in triplicate. Data are represented as the mean ± standard deviation. Different letters indicate statistically significant differences (*p* < 0.05).

On the other hand, the fungal strain produced the highest levels of AME on all three types of media at pH 4.5 after 21 days of incubation, followed by pH 5.5 and 6.5 (Figure 6). Also, the levels of AME production on all three types of media were highest at 25 °C after 21 days. These results are the same as the results of AOH production described above. In addition, the AME production was approximately 3.3-fold higher on SPAM (2.03 ± 0.33 μg/mg dry weight) than YES (0.62 ± 0.01 μg/mg dry weight) under pH 4.5 and 25 °C conditions after 21 days of incubation. In contrast, the AME production on MEA was very low (0.20 ± 0.02 μg/mg dry weight) under the same conditions. The AME production on SPAM was significantly reduced when the incubation temperature increased to 30 °C from 25 °C (Figure 6B,C), which is similar to AOH production on SPAM (Figure 5B,C). These results indicate that the fungal strain produced the highest levels of AME on SPAM under pH 4.5 and 25 °C conditions.

Overall, our data demonstrated that the optimum pH and temperature for the growth of *A. alternata* OM1 were pH 6.5 and 25 °C, respectively, whereas those for its production of AOH and AME were pH 4.5 and 25 °C, respectively. The results also showed that the fungal strain showed the highest growth on YES, while it produced the highest levels of AOH or AME on SPAM after 21 days of incubation.

Thus, because the fungal strain produced the highest levels of AOH and AME on SPAM under pH 4.5 and 25 °C conditions, we chose the pH, temperature, and media as culture conditions for further experiments.

### 3.5. Influence of RH on the Growth of A. alternata OM1 and Its Production of AOH and AME

**Figure 7:**
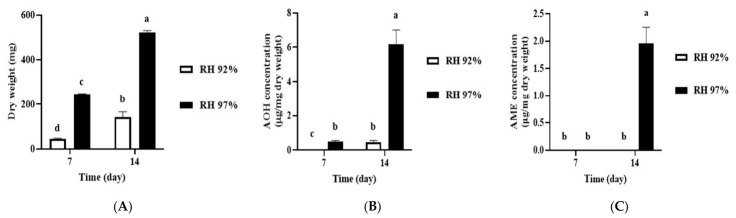
Growth rates of *A. alternata* OM1 and its production of AOH and AME on SPAM (pH 4.5) agar plates under two different RH (92 and 97%) conditions at 25 °C. (**A**) Dry weight of mycelia after 7 and 14 days of incubation, and levels of (**B**) AOH and (**C**) AME after 7 and 14 days of incubation. The levels of AOH and AME, and mycelial dry weight were measured in triplicate. Data are represented as the mean ± standard deviation. Different letters indicate statistically significant differences (*p* < 0.05).

It is known that RH is another abiotic factor that influences fungal growth and mycotoxin production [20,21]. Thus, we cultured *A. alternata* OM1 on SPAM agar plates (pH 4.5) under two different RH conditions (92 and 97%) at 25 °C for 14 days. As expected, the growth pattern of the fungal strain based on its mycelial dry weight was similar to that based on colony diameters on SPAM under both RH conditions over 14 days (Figure 7A and Figure S6). Although the strain showed fast growth rates under RH 97% for 14 days of incubation, it exhibited very slow growth rates under RH 92% and produced approximately 3.7-fold lower dry weight under RH 92% (142.00 ± 24.16 mg) than that under RH 97% (523.10 ± 7.82 mg) on the 14th day (Figure 7A). Also, the fungal strain produced the high level of AOH or AME on SPAM under RH 97% (6.19 ± 0.82 μg/mg dry weight for AOH, 1.96 ± 0.30 μg/mg dry weight for AME) after 14 days although it produced a very low level of AOH or AME on the same media under RH 92% (0.46 ± 0.08 μg/mg dry weight for AOH, not detected for AME) on the same day (Figure 7B). These levels of AOH and AME are consistent with the above results shown in Figure 5B and Figure 6B. Thus, as expected, these results indicate that increased amounts of AOH and AME are produced by *A. alternata* OM1 under the higher percentage of RH (97%) than the lower RH (92%) condition.

When taken together with the above results, our data demonstrated that the three major environmental parameters, including pH, temperature, and RH, significantly influenced the growth of *A. alternata* OM1 and its production of AOH and AME.

### 3.6. Growth Rates of A. alternata OM1 and Its Production of AOH and AME in Three Types of Liquid Media

**Figure 8:**
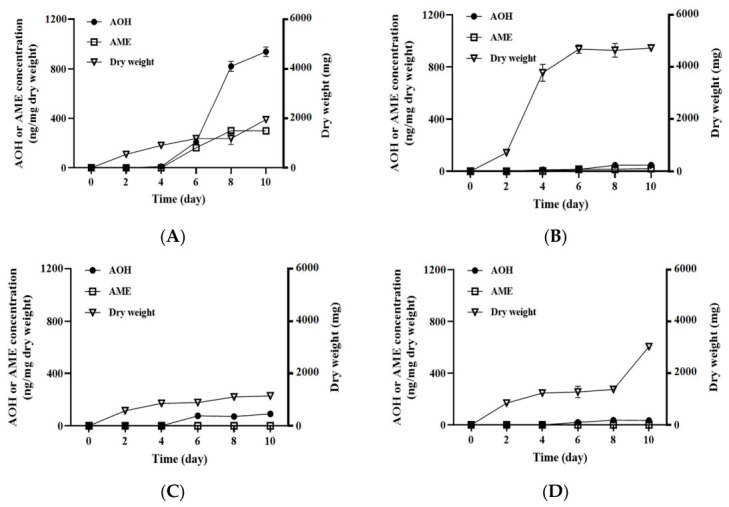
Growth rates of *A. alternata* OM1 and its production of AOH and AME in two different liquid media (YES and MEB) at 25 °C under static or agitation conditions. (**A**) YES under static conditions, (**B**) YES under agitation conditions, (**C**) MEB under static conditions, and (**D**) MEB under agitation conditions. The levels of AOH and AME, and mycelial dry weight were measured in triplicate. Data are represented as the mean ± standard deviation.

As described in Figure 5 and Figure 6, our data exhibited that *A. alternata* OM1 produced the highest, middle, and lowest levels of AOH or AME on SPAM, YES, and MEA agar plates, respectively. However, the strawberry puree liquid media contained many suspended solids such as pulp and seeds, which were derived from strawberries, resulting in the difficult measurement of fungal growth. Thus, in order to compare the amounts of AOH or AME produced by *A. alternata* OM1 in liquid media, the fungal strain was cultured in YES and MEB under no shaking or shaking conditions because it has been known that toxic secondary metabolites such as patulin are produced at higher levels by filamentous fungi under static conditions than agitation conditions [29]. After AOH or AME was not detected until 4 days of incubation, the levels increased in YES under both conditions over 10 days (Figure 8A,B). The AOH level reached the highest (939.11 ± 33.71 ng/mg dry weight) in YES without shaking on the 10th day, whereas it reached the highest (47.81 ± 10.51 ng/mg dry weight) in YES with shaking on the same day (Figure 8A,B). It indicates that the level of AOH in YES without shaking was 20-fold higher than that in YES with shaking. In addition, the AME production showed similar patterns to the AOH production under both culture conditions. After 4 days of incubation, the level of AME increased rapidly in YES without shaking until 10 days of incubation, whereas it increased very slowly in YES with shaking (Figure 8A,B). The level of AME in YES without shaking was 15-fold higher than that in the same media with shaking (298.35 ± 13.02 ng/mg dry weight on the 10th day in static conditions, 20.92 ± 1.03 ng/mg dry weight on the 10th day in agitation conditions). However, the mycelial dry weight in YES without shaking increased moderately over 10 days (1947.00 ± 0.53 mg on the 10th day), while that in YES with shaking increased rapidly (4725.80 ± 32.05 mg on the 10th day) (Figure 8A,B). In contrast to the fungal culture in YES, the fungal strain produced significantly decreased levels of AOH or AME in MEB under both culture conditions (Figure 8C,D). The AOH level in MEB without shaking was approximately 3-fold higher than that in the same media with shaking (91.27 ± 6.22 ng/mg dry weight on the 10th day under static conditions, 31.84 ± 10.97 ng/mg dry weight on the 10th day under agitation conditions). However, AME was not detected in MEB under both culture conditions over 10 days (Figure 8C,D). The mycelial dry weight in MEB exhibited a 2.6-fold higher level under agitation conditions than under static conditions on the 10th day (3028.73 ± 8.20 mg under agitation conditions, 1144.83 ± 3.76 mg under static conditions). Overall, our data demonstrated that levels of AOH and AME produced by *A. alternata* OM1 were highest in YES under static conditions, whereas its dry weight was highest in the same media under agitation conditions among the two types of media (YES and MEB).

### 3.7. Relative Gene Expression Analysis by RT-qPCR

**Figure 9:**
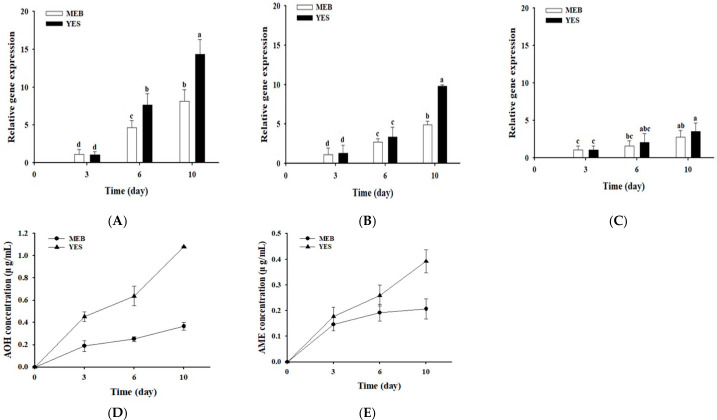
Time course of relative expression levels of AOH or AME biosynthetic genes in *A. alternaria* OM1 and its AOH or AME level in YES (AOH- or AME-supportive) or MEB (AOH- or AME-non-supportive) medium. Relative expression levels of (**A**) *pksI*, (**B**) *omtI*, and (**C**) *aohR*, and levels of (**D**) AOH and (**E**) AME in YES or MEB. The expression levels of three genes and levels of AOH or AME were measured in triplicate. The expression levels were normalized to that of β-tubulin gene. Data are represented as the mean ± standard deviation. Different letters indicate statistically significant differences (*p* < 0.05).

The relative expression levels of three AOH or AME biosynthetic genes (*pksI*, *omtI*, and *aohR*) in the gene cluster were analyzed by RT-qPCR after culture of *A. alternata* OM1 in YES (AOH or AME supportive) or MEB (AOH or AME non-supportive) medium. Of the three genes, the relative expression levels of two AOH or AME biosynthetic genes (*pksI* and *omtI*) increased gradually in both types of media over 10 days of incubation (Figure 9A,B). However, the increases in their expression levels in YES were larger than those in the expression levels in MEB after 10 days. In contrast, the relative expression levels of *aohR* remained relatively constant in both YES and MEB over 10 days, and there was no statistically significant difference between the expression levels in both media (Figure 9C). One of the reasons for this may be attributed to constitutive expression of *aohR* in both types of media, which encodes Zn(II)2Cys6 transcription factor. Additionally, HPLC analyses exhibited that *A. alternata* OM1 produced higher levels of AOH and AME in YES (1.08 ± 0.01 μg/mL for AOH and 0.39 ± 0.05 μg/mL for AME on the 10th day) than those in MEB (0.37 ± 0.04 μg/mL for AOH and 0.21 ± 0.04 μg/mL for AME on the 10th day) (Figure 9D,E), which is in line with the data shown in Figure 8A,D. These results are also consistent with the gene expression patterns by RT-qPCR described above, indicating that the two AOH or AME biosynthetic genes were up-regulated in YES (AOH- or AME-supportive), whereas they were not in MEB (AOH- or AME-non-supportive).

## 4. Discussion

It has been reported that abiotic factors, including temperature, pH, RH, and water activity (a_w_), impact fungal growth and mycotoxins production in food [23,25,42,43]. Two major *Alternaria* mycotoxins, AOH and its derivative AME, are commonly found in food such as cereal grains and fruits [4,5,6]. Most previous studies have been focused on environmental parameters for AOH and AME production by *A. alternata* or *A. arborescens* strains, which were isolated from certain cereal grains and fruits such as soybeans and tomatoes, on synthetic or semi-synthetic media or natural food products [2,3,11,24,25,27,28]. Thus, in the present study, we isolated *A. alternata* OM1 from strawberry jam, which was able to produce AOH and AME, and investigated the effects of the 3 major environmental factors (temperature, pH, and RH) on its growth and production of AOH and AME on natural product media containing strawberry puree.

Our results exhibited that five fungal isolates from strawberry jam belong to *Neurospora* spp. and *Alternaria* spp. One previous study from Denmark reported that the authors isolated mainly *Cladosporium* spp., *Botrytis* spp., *Penicillium* spp., *Aspergillus* spp., and *Alternaria* spp. in strawberries (cultivar Florence) [44], which is in line with our results. Interestingly, our data showed that the five fungal strains (1 *A. alternata* OM1, 2 *Neurospora sitophila*, 1 *Neurospora tetrasperma*, and 1 *Neurospora* sp.) were isolated from strawberry jam samples (homemade products) although they were heated at 80 °C for 20 min after a washing, manufacturing, and packaging steps for the processing of raw strawberries, which followed a standard manufacturing procedure. This may have resulted from insufficient heating during this step. In addition, we verified that *A. alternata* OM1 was able to produce AOH and AME, but not TeA. We attempted to detect TeA that could be produced by the strain in PDB containing 1 or 5% DMSO, since a previous study showed that the addition of DMSO to a medium induced TeA production by *Magnaporthe oryzae* [45]. However, *A. alternata* OM1 was not able to produce TeA even in the media containing DMSO. Previously, it was reported that some *A. alternata* strains were able to produce only AOH and its derivative AME, although many *A. alternata* strains can produce the three major *Alternaria* mycotoxins [46,47]. *A. alternata* strains share a common biosynthetic pathway for simultaneous production of AOH and its derivative AME, while they use a different separate pathway for production of TeA, a tetramic acid derivative [5,6]. Thus, some *A. alternata* strains, including *A. alternata* OM1, may not be able to produce TeA due to a blockage in the TeA biosynthetic pathway or the absence of the biosynthetic genes.

Our data also showed that the optimum temperature and pH for growth of *A. alternata* OM1 were 25 °C and pH 6.5, respectively, and that the fungal strain produced the highest growth on YES among four different media (SPAM, YES, PDA, and MEA). It is in agreement with the data from other previous studies [2,3,26]. In Prendes and co-workers’ study, they described that the optimum temperature for growth of three *A. alternata* strains isolated from wine grapes was 25 °C among three temperature conditions (15, 25, and 30 °C) when they were cultured on synthetic media with a composition similar to grapes [26]. Also, in another study, the authors showed that *A. alternata* isolated from soybeans had the maximal growth rate on soybean extract agar at 25 °C among four temperature conditions (5, 18, 25, and 30 °C) [2]. One study from Argentina documented that the optimum growth temperature for a cocktail of five *A. alternata* strains isolated from tomatoes was 21 °C among four temperature conditions (6, 15, 21, and 35 °C) on tomato-based media [3], which is similar to our data. In addition, it is known that *Alternaria* spp. grow best at room temperature but are also capable of growing at low temperatures, leading to spoilage of fruits and vegetables during refrigerated transport and storage [11]. One study reported that the storage temperature of tomatoes should be maintained below 7 °C and their storage period should not exceed 10 days to control the growth of *A. alternata* and its mycotoxin production [24]. Similarly, in our study, the growth of *A. alternata* OM1 was not detected on any type of media at 5 °C until 7 days. Moreover, our data showed the highest growth rate of *A. alternata* OM1 on YES among four different types of media (SPAM, YES, PDA, and MEA). It may have come from higher sugar content (15% sucrose) in YES than other types of media (approximately 4% glucose and 4% sucrose in strawberries, 2% glucose in PDA, approximately 0.01% glucose in MEA) [48]. Also, a previous study from China described that *A. alternata* ACT-3 isolated from cherries had high growth rates on PDA in the range of pH 6.0–8.0 after 12 days at 28 °C [22]. Another study reported that an *A. alternata* strain isolated from noni leaves exhibited maximum growth in PDB at pH 6.0–6.5 after 10 days at 28 °C, which was followed by pH 7.0, 8.0, 5.5, and 4.5 [49]. These data are in line with our results, in which *A. alternata* OM1 showed the highest growth rate on all three types of media at pH 6.5 after 21 days, which was followed by pH 7.5, 5.5, and 4.5.

Several previous studies have shown that production of AOH and AME by *A. alternata* or *A. arborescens* is significantly affected by incubation temperature [2,11,24,25,26]. In some studies, the optimum temperature for AOH production by *A. alternata* was in the range of 15–25 °C, whereas that for its AME production was in the range of 30–35 °C [2,11,25]. One study documented that a cocktail of five *A. alternata* strains isolated from tomatoes produced the highest level of AOH on tomato-based media at 21 °C among four temperature conditions (6, 15, 21, and 35 °C) under a_w_ 0.954 after 28 days, whereas the cocktail strains produced the highest level of AME at 35 °C under the same conditions [11]. Another study reported that two *A. alternata* strains isolated from soybeans produced the highest amounts of AOH on soybean extract agar at 25 °C among four temperature conditions (5, 18, 25, and 30 °C) under a_w_ 0.98 after 35 days, while the strains produced the highest amounts of AME on the same media at 30 °C under a_w_ 0.92 or 0.94 after 28 days [2]. Oviedo et al. also described slightly different results from those on soybean extract agar when they cultured the two *A. alternata* strains on irradiated soybeans, which produced the highest amounts of AOH and AME at 15 or 25 °C and at 25 °C, respectively, after 21 days [25]. However, other studies documented that the optimum temperature conditions for the production of both AOH and AME were the same [23,24,26]. One study described that *A. alternata* strains, which were isolated from grapes, produced the highest levels of AOH and AME on synthetic media at 25 °C among three temperature conditions (15, 25, and 30 °C) [26]. Interestingly, the level of AOH was higher than that of AME in one *A. alternata* strain at 25 °C, whereas the level of AME was higher than that of AOH in the other two *A. alternata* strains at the same temperature, indicating that production of AOH and AME was strain dependent. Donato et al. reported slightly different results, in which two *A. alternata* strains isolated from chickpeas produced the highest levels of AOH and AME on chickpea-based media at 30 °C among four temperature conditions (4, 15, 25, and 30 °C) [23]. Also, similar results were observed from a study from Egypt, which showed the highest levels of AOH and AME production by *A. alternata* IMI 89344 on tomato-based media at 28 °C [24]. In our study, the highest amounts of AOH and AME were produced by *A. alternata* OM1 on SPAM at 25 °C after 21 days. These data suggest the species-specific impact of temperature on mycotoxin production. Moreover, our data showed that *A. alternata* OM1 produced higher levels of AOH than those of AME on all three different types of media. Although the incubation time is considered, the results from some previous studies are slightly different from those of our study. Also, in our study, the optimal temperature for the production of AOH and AME by *A. alternata* OM1 was the same (25 °C) as that for its growth, which is similar to the results from most previous studies on *A. alternata* [2,26], although the optimal temperatures for the production of other mycotoxins such as patulin or penitrem A were lower than those for their growth [29,50]. Again, one of the possible reasons for these may be due to the use of different *A. alternata* strain.

On the other hand, a previous study showed that acidic conditions (pH 4.0–4.5) were more favorable for the production of AOH and AME by *A. alternata* DSM 12633 on modified Czapek-Dox media than neutral or alkaline conditions (above pH 5.5) [42]. It is in good agreement with our results, which exhibited that *A. alternata* OM1 produced the highest AOH and AME on all three different types of media at pH 4.5, which was followed by pH 5.5 and 6.5. There are plenty of previous studies that described the influence of environmental pH on mycotoxin production [29,43,51]. One study documented that a high level of aflatoxin was produced by an *Aspergillus parasiticus* strain under acidic conditions (pH 4.0–5.0) [43]. Another study reported that *Aspergillus ochraceus* HP produced high amounts of ochratoxin on Czapek-Dox broth supplemented with yeast extract (pH 3.0–4.0) [51]. We also showed in a previous study that high levels of patulin were produced by *Penicillium paneum* OM1 under acidic conditions (pH 4.5–5.0) [29]. It is possible that the production of AOH and AME in this study is regulated by PacC, a pH-dependent global transcription factor for secondary metabolism, which can act as an activator for mycotoxin production under acidic conditions [52].

It has been reported that in general the biosynthesis of secondary metabolites such as mycotoxins by *Penicillium* spp. is significantly enhanced on YES media containing sucrose compared to the media containing other carbon sources such as glucose or fructose [53]. Additionally, in our previous study, we showed that *P. crustosum* OM1 produced approximately 2-fold higher amounts of penitrem A, a mycotoxin, on YES containing 4% sucrose than on the same media containing 15%. [50]. Similarly, in our study, *A. alternata* OM1 produced lower amounts of AOH or AME on YES (15% sucrose) than SPAM (4% sucrose). Also, RT-qPCR analysis data showed that YES containing sucrose supports more amounts of AOH and AME production than MEB containing glucose. However, it is known that higher contents of sugar such as glucose or sucrose support fungal growth by serving as an energy source via the TCA cycle in primary metabolism [50,54]. Our study also showed that the growth of *A. alternata* OM1 was higher on YES than SPAM.

Another environmental factor, RH, also influences fungal growth and mycotoxin production [2,11,26]. Previously, one study reported that AOH and AME were produced by an *A. alternata* strain on synthetic media under a_w_ above 0.96 after 21 days at 25 °C, although the strain grew at a_w_ 0.95 under the same conditions [26]. Another study from Argentina described that a cocktail of *A. alternata* strains produced both AOH and AME on tomato-based media at a_w_ 0.954 after 14 days at 21 or 35 °C, but produced only a small amount of AME at a_w_ 0.922 under the same conditions [11]. Similarly, Oviedo et al. documented that they detected AOH and AME production by one *A. alternata* strain on soybean extract agar at 25 °C under a_w_ 0.92 after 21 days, while they did not detect the production of both mycotoxins by the other *A. alternata* strain on the same media under the same conditions [2]. Our data are slightly different from those of the previous studies. In our study, we showed that *A. alternata* OM1 produced high growth rates and increased levels of AOH and AME under the higher percentage of RH (97%) than lower RH (92%) conditions, and that the strain did not produce both mycotoxins under RH 92% until 7 days at 25 °C. These results suggest strain-dependent variability in the ability of *A. alternata* to produce AOH or AME under similar a_w_ or RH conditions. Furthermore, in general, it is known that the minimum a_w_ for growth of *A. alternata* in different media was between 0.84 and 0.88, while a limiting a_w_ for major mycotoxin production by the fungal species lies in the range of 0.88–0.90 [3,11], indicating that the limiting a_w_ for mycotoxin production is slightly higher than that for its growth. It is in good agreement with our results. Overall, when taken together with the above results, our data demonstrated that three major environmental parameters, such as temperature, pH, and RH, had a significant impact on the growth of *A. alternata* OM1 and its production of AOH and AME. Also, our data suggest that storage of strawberries at 5 °C under RH below 92% may pose a low risk of contamination with *A. alternata* OM1 and its mycotoxins at least until 7 days. Previously, one study reported that 24 strawberry samples collected from local markets in Spain contained relatively high levels of AOH and AME (8–752 ng/g for AOH, 8–26 ng/g for AME) [55]. Since currently no data are available for the effects of environmental parameters on the production of AOH and AME by *A. alternata* on strawberry-based media in the literature, knowledge about the environmental conditions could contribute to the control of its growth and production of both mycotoxins on strawberries.

In addition, as expected, our results showed that *A. alternata* OM1 produced the highest level of AOH and AME in YES under static conditions, whereas it had the highest level of dry weight in YES under agitation conditions among two different liquid media (YES and MEB). It is likely that the increased production of both mycotoxins by *A. alternata* OM1 was supported by sucrose in YES as described above and that increased oxygen supply to the fungal cells under agitation conditions led to the increased cell mass but not AOH or AME production. This would have been attributed to the fact that in fungal cells, after conversion of sucrose to glucose, increased amounts of its metabolite acetyl CoA enters into TCA cycle instead of its reaction with malonyl CoA for AOH or AME biosynthetic pathway under agitation conditions, and that the further metabolism of acetyl CoA to electron transport pathway produces ATP as an energy, which is used for fungal growth [56]. These results are similar to those from earlier studies on most other mycotoxins, including patulin and penitrem A as secondary metabolites [29,50].

## 5. Conclusions

In the current study, we investigated the influence of three major environmental factors (temperature, pH, and RH) on the growth of *A. alternata* OM1 isolated from strawberry jam and its production of AOH and AME. Our results demonstrated that the environmental parameters significantly influenced both the growth of the fungal strain and its mycotoxin production. It showed that *A. alternata* OM1 produced the highest growth rate at 25 °C under pH 6.5 and RH 97% conditions, while it produced the highest amounts of AOH and AME at 25 °C under pH 4.5 and the same RH conditions. Moreover, we showed that the fungal strain did not grow on SPAM at 5 °C and did not produce AOH and AME on the same media at 25 °C under RH 92% until 7 days, which are marginal or submarginal temperature and RH conditions for fungal growth and its production of AOH and AME on the media containing strawberries. Thus, our data suggest that refrigeration of strawberries below 5 °C for up to 7 days could prevent the growth of *A. alternata* OM1 and its mycotoxin production on them. Moreover, our data showed that *A. alternata* OM1 was not able to produce TeA. It would be necessary to analyze TeA biosynthetic genes in the fungal strain and to confirm the limiting environmental conditions against its growth and mycotoxin production on strawberries in future studies.

## Figures and Tables

**Table 1:** Primer sequences for RT-qPCR analysis of three AOH or AME biosynthetic genes.

Gene	Orientation	Primer Sequence
*pksI*	Forward	5′ GAGTGATGCTCAATCGCTTC 3′
	Reverse	5′ CTTGATGTGCCTTGGACTTG 3′
*omtI*	Forward	5′ TTGGATGAGTACCCCATTCG 3′
	Reverse	5′ CTTGAACATCGCGCAGTAAT 3′
*aohR*	Forward	5′ CAGACAAGAGATGCGTTCG 3′
	Reverse	5′ GAACGGCTACTTCAAACCTT 3′
*β-tubulin*	Forward	5′ GCGCATGAACGTCTACTTCA 3′
	Reverse	5′ GGCACGAACTTGTTGTTGGA 3′

## Data Availability

The original contributions presented in this study are included in the article/Supplementary Material. Further inquiries can be directed to the corresponding author.

## Supplementary Materials

The following supporting information can be downloaded at: https://www.mdpi.com/article/10.3390/jof12050303/s1, Figure S1: Calibration curves of three different types of mycotoxin standard solutions for quantification of (A) AOH, (B) AME, and (C) TeA by HPLC. A series of AOH or AME standard solutions were prepared in the range of 0.1, 0.2, 0.5, 1.0, and 2.0 μg/mL, whereas TeA standard solutions were prepared in the range of 0.5, 1.0, 2.0, 5.0, and 10.0 μg/mL Each solution was injected into HPLC-UVD in triplicate; Figure S2: Morphology of *A. alternata* OM1 on four different culture media. (A) Fungal colonies on PDA agar plates, (B) fungal colonies on SPAM agar plates, (C) fungal colonies on YES agar plates, (D) fungal colonies on MEA agar plates, (E) conidiophores and conidiospores on PDA agar plates (400×), and (F) conidiophores and conidiospores on MEA agar plates (400×). Left photographs show top views, whereas right photographs show bottom views in (A), (B), (C), and (D). Scale bar, 100 μm; Figure S3: Extracted ion chromatograms (EIC) and mass (MS) spectra of AOH and AME. (A) EIC and (B) MS spectrum for AOH in an AOH standard solution (2 μg/mL), (C) EIC and (D) MS spectrum for AOH in culture extracts of *A. alternata* OM1, (E) EIC and (F) MS spectrum for AME in an AME standard solution (2 μg/mL), and (G) EIC and (H) MS spectrum for AME in culture extracts of *A. alternata* OM1. (Inset) AOH structure in (B) and AME structure in (F); Figure S4: Growth of *A. alternata* OM1 on four types of solid media (SPAM, PDA, YES, and MEA) under four different temperature conditions (5, 20, 25, and 30 °C). (A) Colony diameter on SPAM, (B) colony diameter on PDA, (C) colony diameter on YES, and (D) colony diameter on MEA. The mycelial colony diameter was measured in triplicate. Data are represented as the mean ± standard deviation; Figure S5: Growth of *A. alternata* OM1 on three types of solid media (SPAM, YES, and MEA) under four different pH conditions (pH 4.5, 5.5, 6.5, and 7.5) at 25 °C. (A) Colony diameter on SPAM at 25 °C, (B) colony diameter on YES at 25 °C, and (C) colony diameter on MEA at 25 °C. The mycelial colony diameter was measured in triplicate. Data are represented as the mean ± standard deviation; Figure S6: Growth of *A. alternata* OM1 on SPAM (pH 4.5) agar plates under two different RH (92 and 97%) conditions at 25 °C. The mycelial colony diameter was measured in triplicate. Data are represented as the mean ± standard deviation. Different letters indicate statistically significant differences (*p* < 0.05); Table S1: Levels of AOH and AME produced by fungal isolates and their scientific names identified by BLASTn-based analysis.

## Abbreviations

The following abbreviations are used in this manuscript:

AOH	Alternariol
AME	Alternariol monomethyl ether
TeA	Tenuazonic acid
ITS	Internal transcribed spacer
SPAM	Strawberry puree agar medium
RH	Relative humidity
