# Supplementary material for: Effects of Key Environmental Factors on Growth of Alternaria alternata Isolated from Strawberry Jam and Its Production of Alternariol and Alternariol Monomethyl Ether
Source: J Fungi (Basel). 2026 Apr 22;12(5):303. doi: 10.3390/jof12050303 (PMC13207730; doi:10.3390/jof12050303)
Supplement: Supplementary file 1 [file jof-12-00303-s001.zip › jof-4240315-supplementary.pdf]

Supplementary Figures

A.

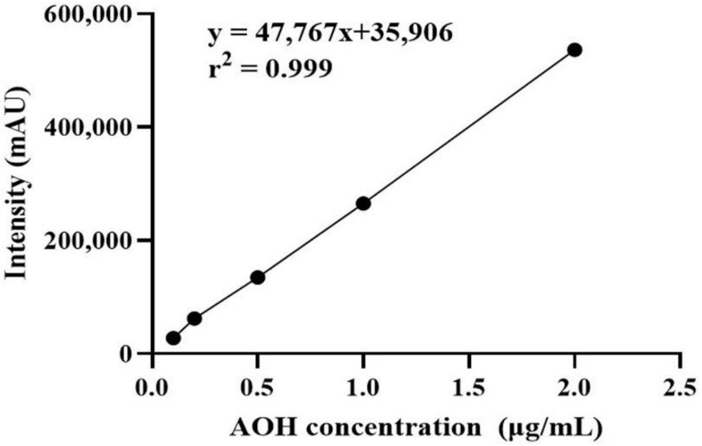

B.

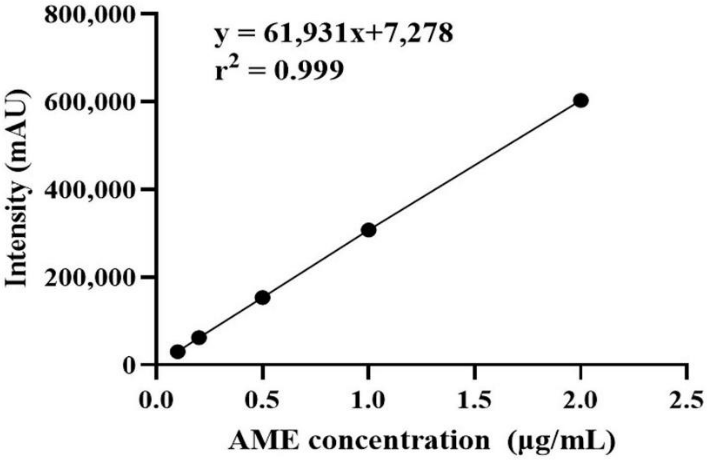

C.

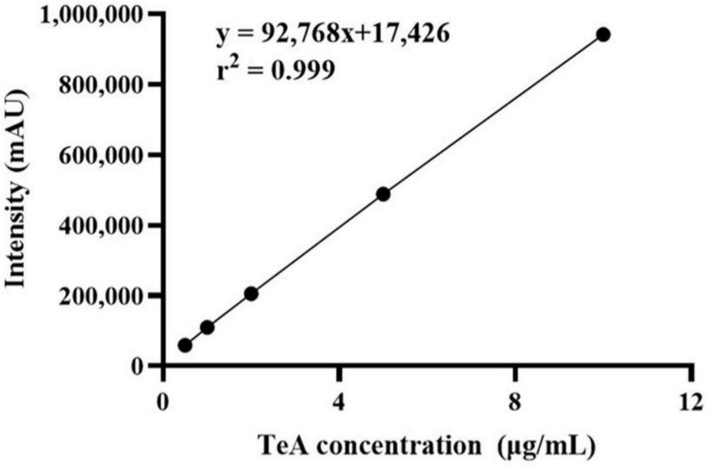

Figure S1. Calibration curves of three different types of mycotoxin standard solutions for quantification of (A) AOH, (B) AME, and (C) TeA by HPLC. A series of AOH or AME standard solutions were prepared in the range of 0.1, 0.2, 0.5, 1.0, and 2.0 μg/mL, whereas TeA standard solutions were prepared in the range of 0.5, 1.0, 2.0, 5.0, and 10.0 μg/mL Each solution was injected into HPLC-UVD in triplicate.

A.

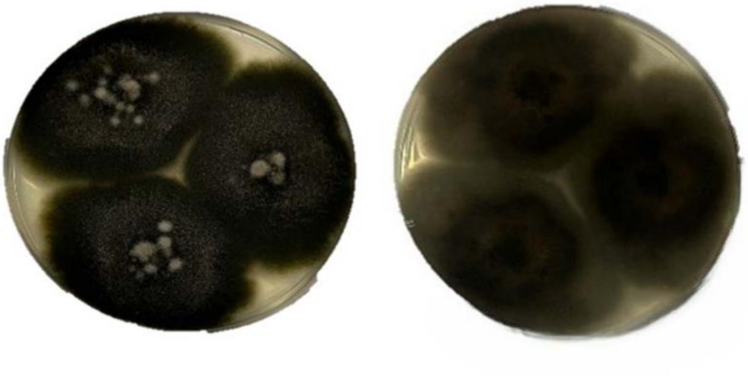

B.

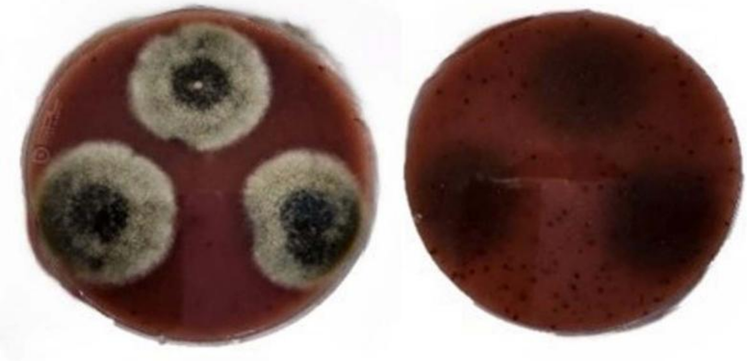

C.

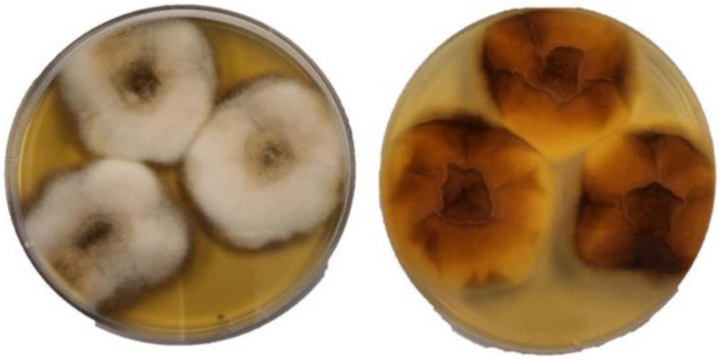

D.

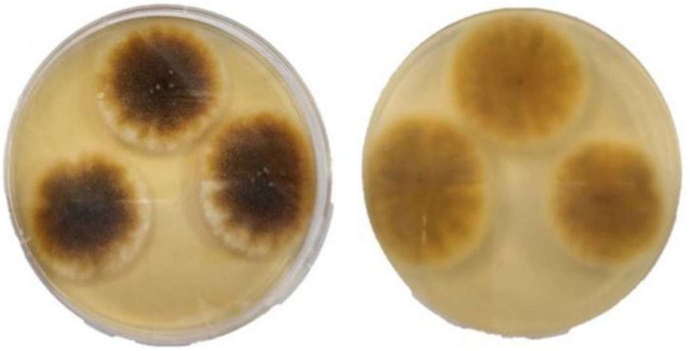

E.

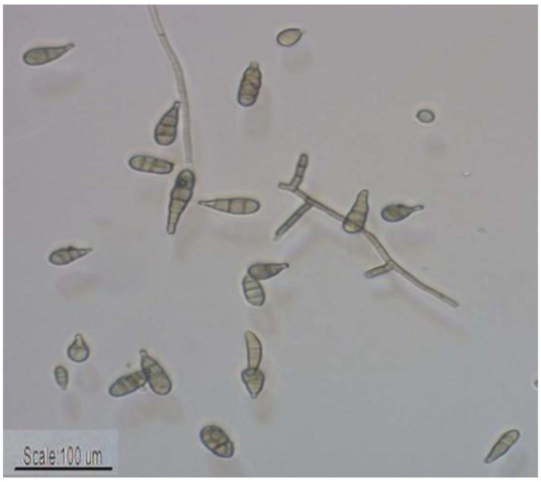

F.

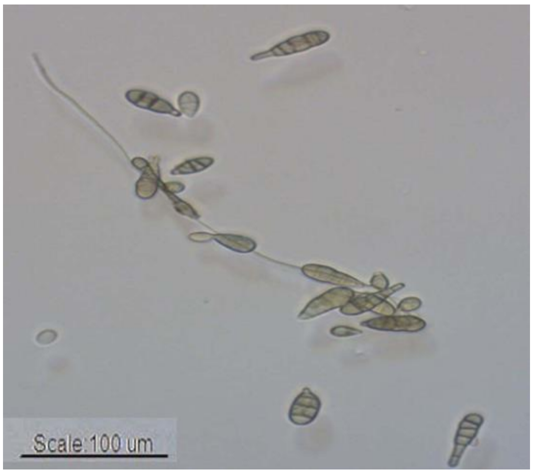

Figure S2. Morphology of *A. alternata* OM1 on four different culture media. (A) Fungal colonies on PDA agar plates, (B) fungal colonies on SPAM agar plates, (C) fungal colonies on YES agar plates, (D) fungal colonies on MEA agar plates, (E) conidiophores and conidiospores on PDA agar plates (400x), and (F) conidiophores and conidiospores on MEA agar plates (400x). Left photographs show top views, whereas right photographs show bottom views in (A), (B), (C), and (D). Scale bar, 100 μm.

A.

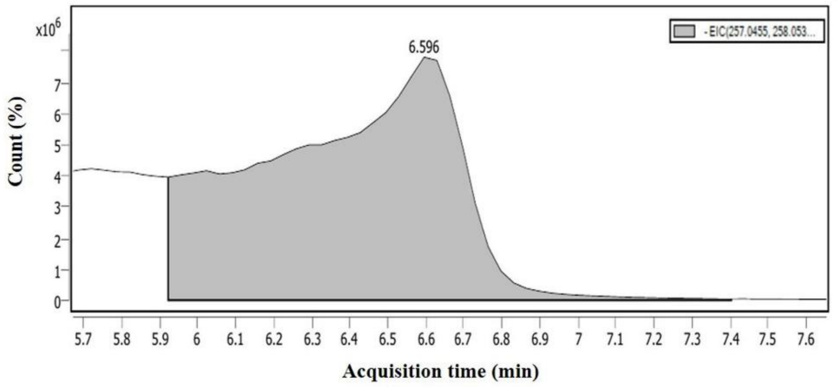
x10^6
6.596
- EIC(257.0455, 258.053...
Count (%)
Acquisition time (min)

B.

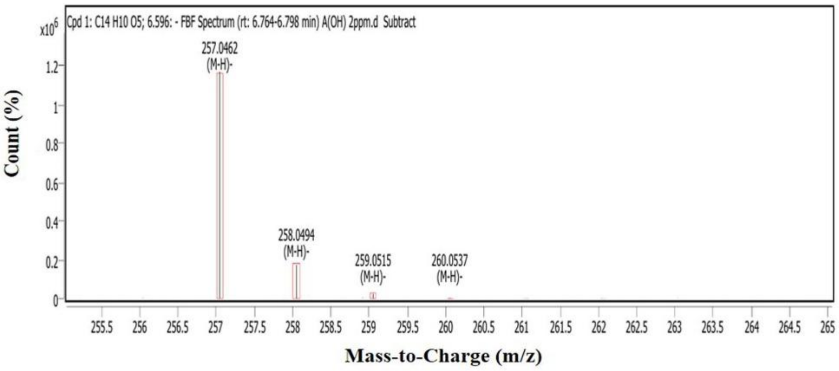
Cpd 1: C14 H10 O5; 6.596: - FBF Spectrum (rt: 6.764-6.798 min) A(OH) 2ppm.d Subtract
x10^6
257.0462
(M-H)-
258.0494
(M-H)-
259.0515
(M-H)-
260.0537
(M-H)-
Count (%)
Mass-to-Charge (m/z)

C.

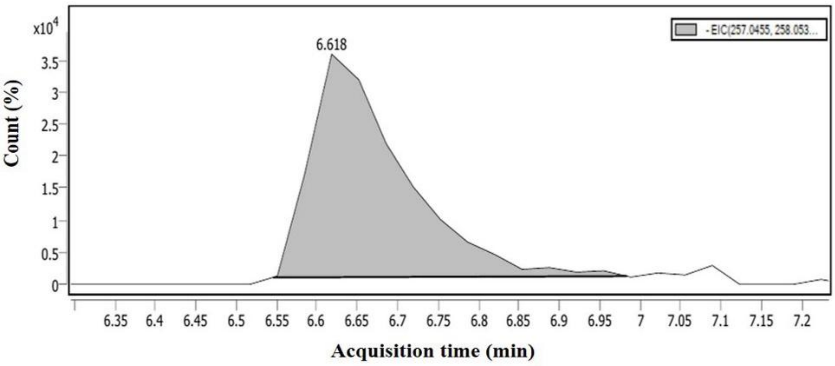
x10^4
6.618
- EIC(257.0455, 258.053...
Count (%)
Acquisition time (min)

D.

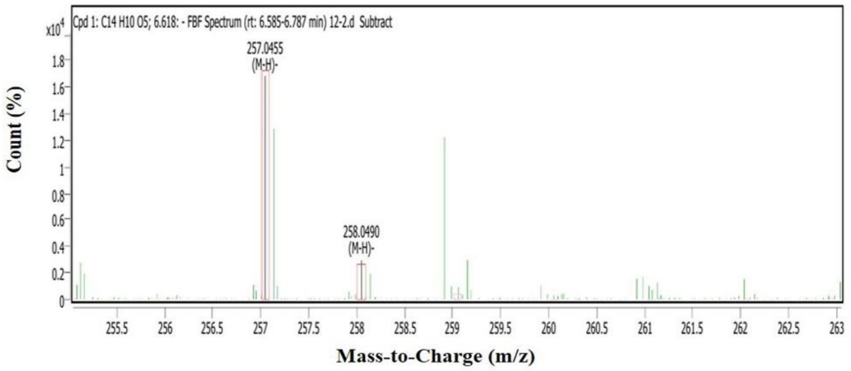
Cpd 1: C14 H10 O5; 6.618: - FBF Spectrum (rt: 6.585-6.787 min) 12-2.d Subtract
x10^4
257.0455
(M-H)-
258.0490
(M-H)-
Count (%)
Mass-to-Charge (m/z)

E.

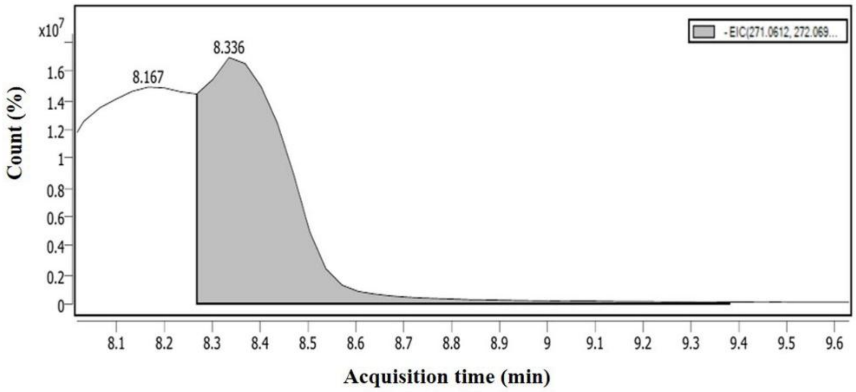
x10^7
- EIC(271.0612, 272.069...
8.167
8.336
Count (%)
Acquisition time (min)

F.

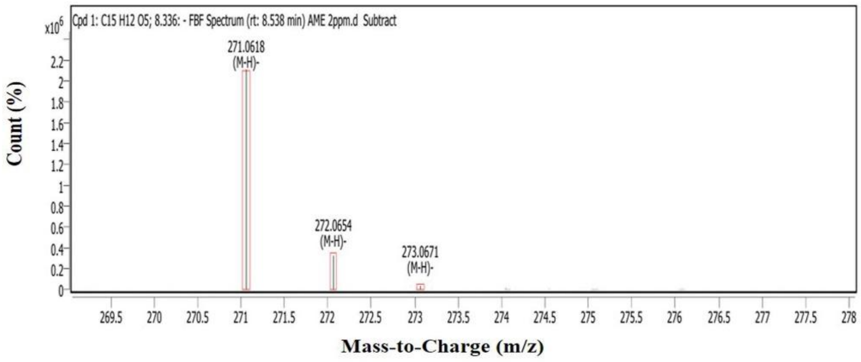
Cpd 1: C15 H12 O5; 8.336: - FBF Spectrum (rt: 8.538 min) AME 2ppm.d Subtract
x10^6
271.0618
(M-H)-
272.0654
(M-H)-
273.0671
(M-H)-
Count (%)
Mass-to-Charge (m/z)

G.

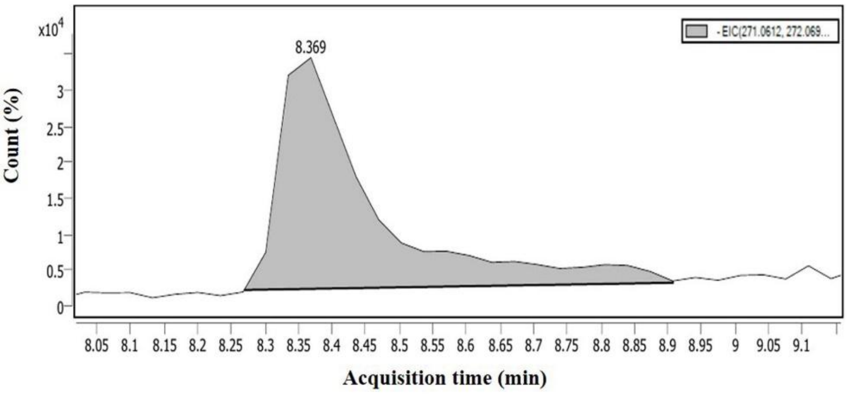

H.

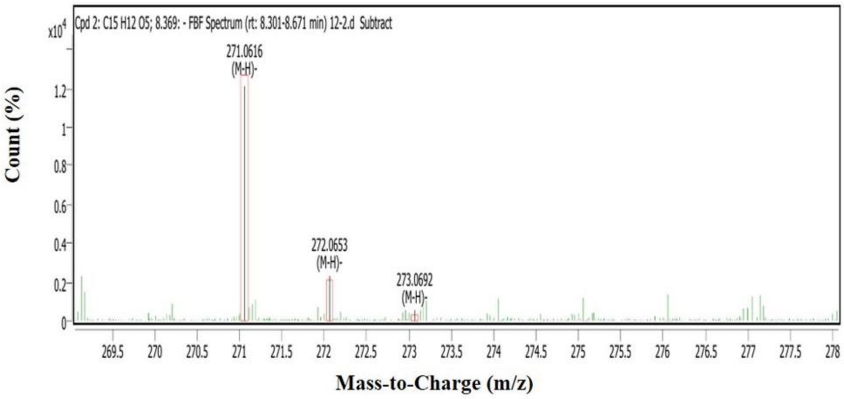

Figure S3. Extracted ion chromatograms (EIC) and mass (MS) spectra of AOH and AME. (A) EIC and (B) MS spectrum for AOH in an AOH standard solution (2 µg/mL), (C) EIC and (D) MS spectrum for AOH in culture extracts of *A. alternata* OM1, (E) EIC and (F) MS spectrum for AME in an AME standard solution (2 µg/mL), and (G) EIC and (H) MS spectrum for AME in culture extracts of *A. alternata* OM1. (Inset) AOH structure in (B) and AME structure in (F).

A.

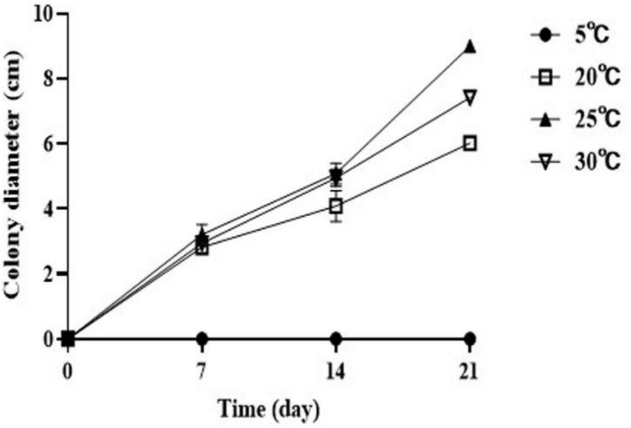

10
8
6
4
2
0
Colony diameter (cm)
0
7
14
21
Time (day)
5℃
20℃
25℃
30℃

B.

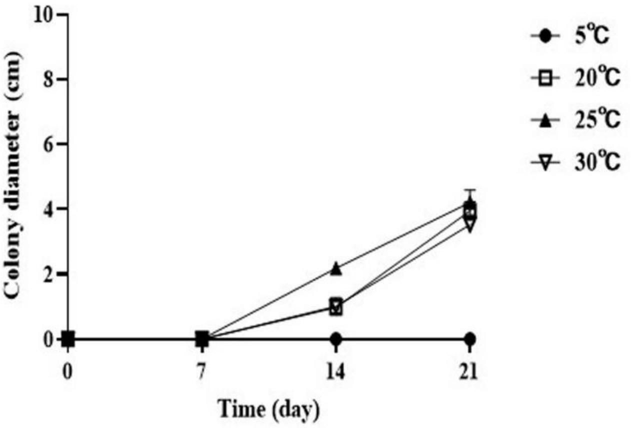

10
8
6
4
2
0
Colony diameter (cm)
0
7
14
21
Time (day)
5℃
20℃
25℃
30℃

C.

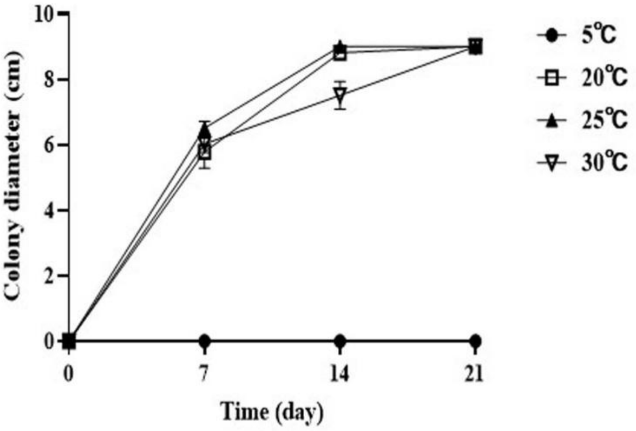

D.

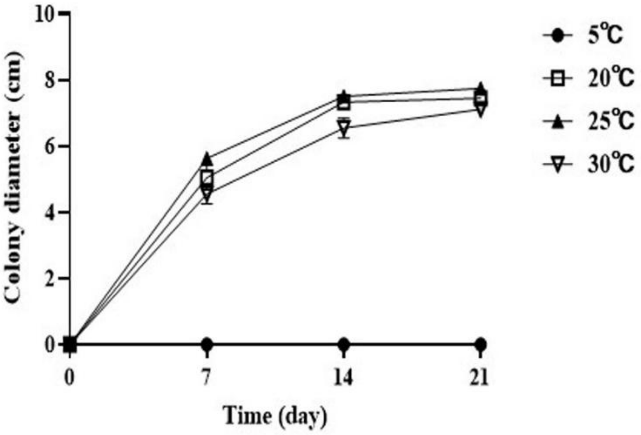

Figure S4. Growth of *A. alternata* OM1 on four types of solid media (SPAM, PDA, YES, and MEA) under four different temperature conditions (5, 20, 25, and 30°C). (A) Colony diameter on SPAM, (B) colony diameter on PDA, (C) colony diameter on YES, and (D) colony diameter on MEA. The mycelial colony diameter was measured in triplicate. Data are represented as the mean ± standard deviation.

A.

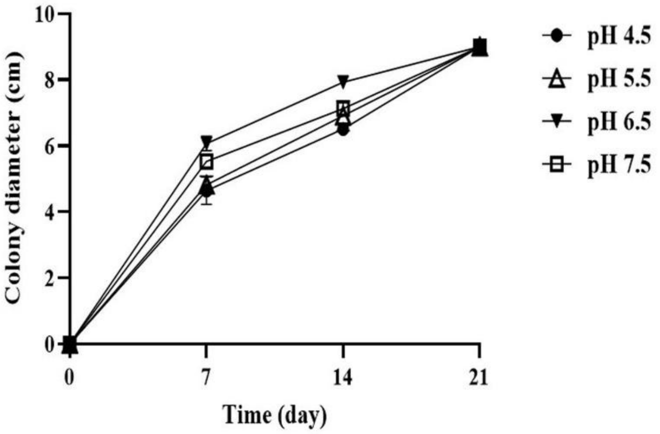

10
8
6
4
2
0
Colony diameter (cm)
0
7
14
21
Time (day)
pH 4.5
pH 5.5
pH 6.5
pH 7.5

B.

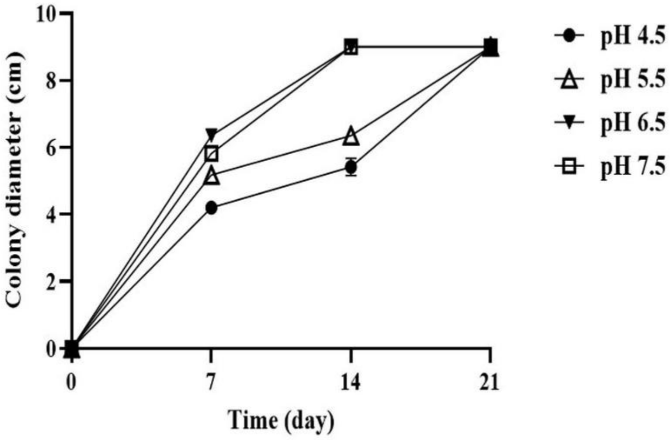

10
8
6
4
2
0
Colony diameter (cm)
0
7
14
21
Time (day)
pH 4.5
pH 5.5
pH 6.5
pH 7.5

C.

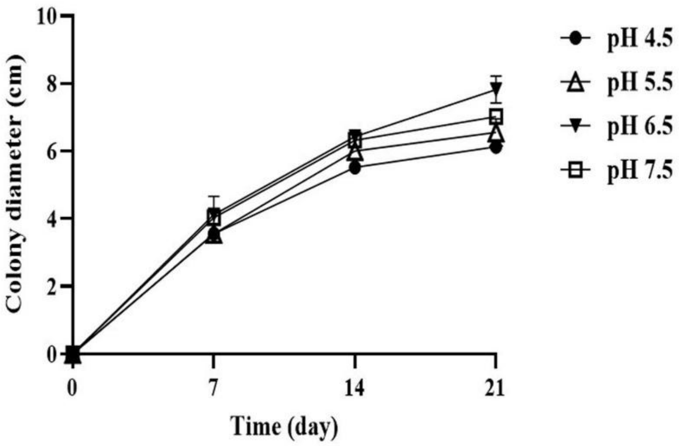

Figure S5. Growth of *A. alternata* OM1 on three types of solid media (SPAM, YES, and MEA) under four different pH conditions (pH 4.5, 5.5, 6.5, and 7.5) at 25°C. (A) Colony diameter on SPAM at 25°C, (B) colony diameter on YES at 25°C, and (C) colony diameter on MEA at 25°C. The mycelial colony diameter was measured in triplicate. Data are represented as the mean ± standard deviation.

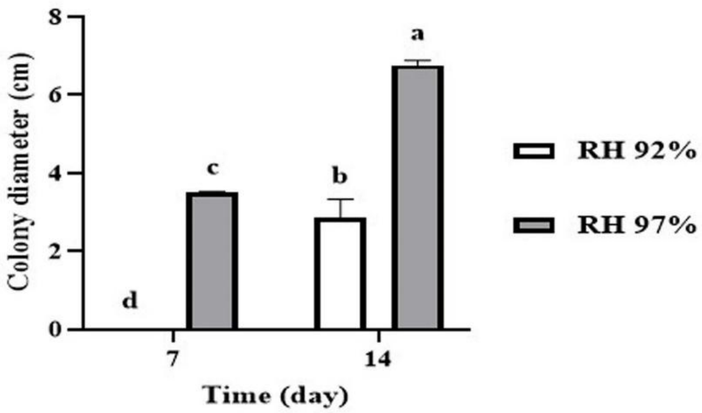

Figure S6. Growth of *A. alternata* OM1 on SPAM (pH 4.5) agar plates under two different RH (92 and 97%) conditions at 25°C. The mycelial colony diameter was measured in triplicate. Data are represented as the mean ± standard deviation. Different letters indicate statistically significant differences ($P<0.05$).

Supplementary Tables

Table S1. Levels of AOH and AME produced by fungal isolates and their scientific names identified by BLASTn-based analysis.

**Sample ID**	**Isolate or strain no. from NCBI**	**Scientific name (BLASTn accession no.)** [1]	**AOH (μg/mL)**	**AME (μg/mL)**
A1	3396	*Neurospora* sp. (FJ527871.1)	ND [2]	ND
A2	Isolate N14R11	*Alternaria alternata* (MT134991.1)	4.67 ± 0.44	1.33 ± 0.14
A3	Isolate UWR 004	*Neurospora tetrasperma* (KX247295.1)	ND	ND
A4	Strain M21	*Neurospora sitophila* (KM588213.1)	ND	ND
A5	Strain M21	*Neurospora sitophila* (KM588213.1)	ND	ND

[1]BLASTn was run using ITS1-5.8S rDNA-ITS2 sequences. BLASTn indicates basic local alignment search tool for nucleotide, while ITS represents internal transcribed spacer.

[2]ND represents not detected.
